# Differentiation of *Vespa velutina nigrithorax* Colonies Using Volatile Organic Compound Profiles of Hornets and Nests

**DOI:** 10.3390/insects15100811

**Published:** 2024-10-16

**Authors:** Omaira de la Hera, Rosa María Alonso

**Affiliations:** FARMARTEM Group, Department of Analytical Chemistry, Faculty of Science and Technology, University of the Basque Country (UPV/EHU), Barrio Sarriena, s/n, 48940 Leioa, Biscay, Spain; omaira.delahera@ehu.eus

**Keywords:** *Vespa velutina nigrithorax*, volatile organic compound profiles, differentiation, hornets, nest

## Abstract

**Simple Summary:**

*Vespa velutina* is a eusocial insect accidentally introduced in Europe (2004) and has been expanded throughout the continent, causing enormous damages in the beekeeping, agriculture, and health sectors. In this work, the profiles of volatile organic compounds of *V. velutina* hornets from four colonies placed in different localities of Biscay (Spain) and from the external cover of their corresponding nests were obtained. Hornets and nests were extracted with hexane and an acetone/methanol mixture (50:50 *v*/*v*) and analysed by a gas chromatography–mass spectrometry (GC-MS) analytical technique. The volatile organic compounds (VOCs) were identified from the profiles obtained and then processed using chemometric tools. These profiles were able to differentiate and discriminate between the different colonies. Furthermore, they allowed us to observe similarities in colonies close in location. The compounds found in common have a great relevance since they could be applied to the development of more efficient control methods for this invasive species based on chemical signals using attractive traps or baits containing the relevant compounds.

**Abstract:**

*Vespa velutina* (Lepeletier, 1836) (Hymenoptera: Vespidae) is a eusocial insect that lives in colonies of hundreds to thousands of individuals, which are divided into castes according to their task: queens, workers, and males. The proper functioning of the colony requires communication between the individuals that make up the colony. Chemical signals (pheromones) are the most common means of communication used by these insects to alarm and differentiate between individuals belonging or not to the colony. In this work, profiles of volatile organic compounds were obtained from the hornets and the external cover of four secondary nests located in the Basque Country. The obtained profiles were treated using chemometric tools. The grouping of hornets and nests according to the different colonies and geographical location was observed. In total, 37 compounds were found in common in hornets and nests. Most of them have been reported in the literature as belonging to different insects and plant species. This would corroborate the transfer of chemical compounds between the nest and the hornets’ nest and vice versa. This information could be applied to the development of more efficient control methods for this invasive species, such as attractive traps or baits containing the relevant compounds.

## 1. Introduction

Insects can be classified according to their level of social behaviour, from solitary to eusocial [1]. In the first group, in which most of insects are found, each individual lives for itself and only interacts with others to mate and lay eggs [2]. In contrast, insects that have some social behaviour, independent of sexual behaviour, are social species. This group has different degrees of sociability, where eusocial insects show the most developed social behaviour, including some species of bees, hornets, wasps, ants, and termites [1,3]. This group of insects lives in large colonies, usually monospecific, consisting of hundreds to thousands of individuals, organised into castes, with different assigned functions. Among the hornets, the invasive hymenopteran *V. velutina nigrithorax* (Lepeletier, 1836) (*V. velutina*) belongs to this group. The caste system of *V. velutina* is divided into the queen, workers, and males [4,5]. This species was accidentally introduced in Europe (2004) and has been expanded throughout the continent, causing enormous damages in the beekeeping, agriculture, and health sectors [6,7,8,9].

The effective performance of colony functions requires communication and identification between the individuals that constitute the colony [10,11]. Insects use different ways of communication, such as acoustic and vibratory, visual, or tactile messages. However, the most used communication is by means of chemical signals (or pheromones) [12], which are secreted by the different glands that hornets possess (abdominal and mouthparts, legs, and antennae) [2,10,13]. These signals are commonly employed by eusocial insects to indicate their presence and/or fertility by the queen to workers; recognition of nestmates from those of other colonies; or as alarm signals to recruit nestmates as a defence [14]. 

One of the most studied families of compounds that have been identified as communication chemicals are the lipids. In their composition are included long-chain aliphatic compounds that vary in size from 27 to 47 carbon atoms. Within them are linear and branched hydrocarbons (HCs), which are usually mono-, di-, and/or trimethylalkanes, as well as unsaturated hydrocarbons with double bonds in different positions, alkyl esters, sterols, glycerides, free alcohols, aldehydes, and free fatty acids [3,15,16]. These can be secreted by the venom and Dufour’s glands (DG). The latter is a small exocrine gland located near the first. These secreted compounds are used as alarm and recognition signals. Additionally, the hydrocarbons are also found in the cuticle and are called cuticular hydrocarbons (CHCs) [14,17]. One of the main functions of these CHCs is to prevent and minimise desiccation of insects, but they also have a recognition function between nestmates and those of other colonies [3,18,19]. 

In general, chemicals on the cuticle are mainly endogenous and can be transferred between workers, as well as from worker to the nest, both by contact and in the construction of nest. The presence of recognition chemicals on exposed nests allows foragers to perceive the odour to return to the colony and repel non-nestmates [18,19]. However, exogenous compounds that are present in the nest building material, or in food, can be absorbed by the cuticle of the insects, giving them a characteristic odour of the colony [19,20,21]. 

This transfer between the individuals of a colony and the nest and vice versa was previously studied by several authors in other species of social wasps and hornets (*Polistes exclamans* (Viereck, 1906), *Vespa crabro* (Linnaeus, 1758), *Polisted fuscatus* (Fabricius, 1793), *Polistes metricus* (Say, 1831), *Polistes biglumis* (Linnaeus, 1758). In these studies, authors observed that the compounds that were identified in the nests were identical to those found in the insect cuticle. 

Consequently, some species of social hornets/wasps have colony-specific CHC profiles [16,19,22]. This fact was demonstrated by Tokoro et al. [22] among other authors [18,23,24,25] by discriminant analysis, who observed how HCs profiles among colonies of the Japanese hornet, *Vespa analis* (Fabricius, 1775) varied. 

Although the importance of lipids as recognition signals has been previously studied in different social insects, the study of these compounds as discriminating compounds of the different colonies of *V. velutina* has not been carried out to date.

Therefore, the aim of this work was to obtain the volatile organic compounds (VOCs) profile of *V. velutina* hornets from four colonies placed in different localities of Biscay (Spain) and from the external cover of their corresponding nests. For this purpose, the hornets and nests were extracted with hexane and an acetone/methanol mixture (50:50 *v*/*v*) and analysed by the GC-MS analytical technique. The VOCs were identified from the volatile profile obtained and then processed using chemometric tools.

The identified compounds in the hornets and in the external cover of the nests widen the knowledge about the colony recognition capacity of this invasive species, as well as on the differentiation of the colonies. Furthermore, those in common give rise to relevant information to know the possible transference between the construction of the nest material and hornets or vice versa. In addition, the recognition compounds in common between colonies could be used to improve the existing control methods, producing more specific attractant traps or baits.

## 2. Materials and Methods

### 2.1. Sample Collection

**Figure 1:**
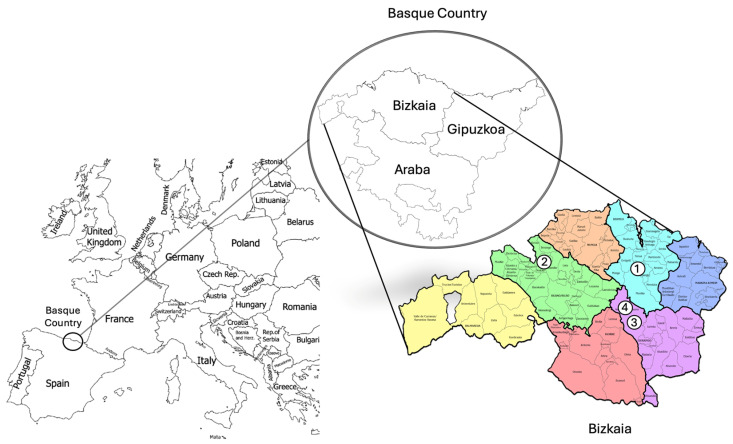
Location of the Basque Country in Europe (left). Map of the Basque Country with its three counties (above) and Bizkaia (right). The numbers indicate the locations of the *Vespa velutina* collected nests in Bizkaia: Ajangiz (1), Leioa (2), and Amorebieta (3 and 4). Image modified from paintmaps.com, accessed on 9 July 2024, and Unai Garcia© (2019).

Four secondary nests from Ajangiz, Leioa, and two from Amorebieta (Biscay, Basque Country, Spain), were collected by specialised personnel in nest removal (Figure 1). The nests were supplied in plastic boxes with holes in the lid, allowing hornets to breathe. Hornets were put to sleep with diethyl ether (99.7%) (Panreac Applichem, Barcelona, Spain) as an anaesthetic, which was added progressively. Then, hornets and the external cover of the nest were divided into two groups based on the polar and non-polar solvent extractions, transferred to storage boxes, and frozen (−20 °C) until their analysis by GC-MS, (Santa Clara, CA, USA). The number of individuals extracted by the two types of extractions was 20 and 5 samples of external cover of the nest for each colony.

### 2.2. Volatile Organic Compounds Extraction

Each whole hornet and the external cover of the nests were weighed (mean weight 0.2513 g and 0.2433 g, respectively) in an analytical balance (precision 0.0001 g) Sartorius CP224S (Madrid, Spain) and were placed in a 10 mL test tube and crushed with a glass rod. For hornets, liquid nitrogen (Air Liquid, Paris, France) was added in order to facilitate the homogenisation of the samples. Then, 2 mL of corresponding extraction solvent was added, and a manual extraction was performed for 1 min using a glass rod. Hexane (Scharlau, Sentmenat, Spain) and a 50:50 (*v*/*v*) mixture of acetone (Merck, Darmstadt, Germany) and methanol (Scharlau, Sentmenat, Spain) (Ac:MeOH), all of them HPLC grade, were used as extraction solvents. The mix was centrifuged (5000 rpm, 10 min) in a 5804 centrifuge from Eppendorf (Hamburg, Germany), and the supernatant was transferred to another test tube. The extraction process was repeated twice on the solids but with the addition of 1.5 mL of the corresponding solvent. The extracts were pooled and dried using a TurvoVap^®^ evaporator (Zymarl, Hopkinton, MA, USA) at 40 °C with a nitrogen gas stream. Finally, the residue was reconstituted in 1 mL of the corresponding solvent to preconcentrate, filtered (0.45 µm), transferred to a 2 mL vial, and injected into the GC-MS system. 

### 2.3. Volatile Organic Compounds Analysis by GC-MS

An Agilent 6890N Network system gas chromatography coupled to a CTC-PAL 120 autosampler (Zwingen, Switzerland) was employed for the analysis of VOCs in hornets and the external cover of the nests. The chromatographic separation was carried out using a HP-5MS UI column (30 m × 0.25 mm ID × 0.25 µm) from Agilent Technologies. An Agilent 5973-N mass spectrometric detector (Santa Clara, CA, USA) coupled to the chromatographic system was used. Table 1 shows the GC-MS analysis conditions.

### 2.4. Data Treatment

#### 2.4.1. Volatile Organic Compound Identification

Volatile organic compounds (VOCs) and their relative concentration in the hornets and their external cover of the nest were identified using the PARADISe software (6.0.1) developed by the University of Copenhagen [26]. It is a tool based on the PARAllel FACtor 2 (PARAFAC2) analysis, and it is used for alignment, deconvolution, and identification of chromatographic peaks from GC-MS data. The NIST14 library database from Agilent Technologies was used to identify the volatile organic compounds. 

Only compounds with more than 70% agreement with the NIST14 library were considered compounds of interest.

#### 2.4.2. Multivariate Analysis

The chromatographic peaks areas of the total compounds identified in hornets and the external cover of the nest by GC-MS were used for the multivariate analysis. Prior to the analysis, in order to avoid biases for the different samples, the obtained data were normalised with the weight. Then, to stabilise the variance of the obtained areas, they were transformed into the base 10 logarithm (log 10) and scaled with mean-centring scaling, which centres data around zero, making easier the comparison between variables and improving the results interpretation in the different multivariate analysis.

In order to find differences and/or groups, as well as possible outliers, a principal component analysis (PCA) was applied to the obtained data in the GC-MS analysis of hornets and the external cover of the nest in the different extraction solvents. 

To assess the importance of the different volatile organic compounds regarding the discriminant classes and remove those without relevance, a Partial Least Squares Discriminant Analysis (PLS-DA) model was built. 

However, for integrating the data obtained in both extraction solvents into a global analysis, multiblock modelling (MB-PCA and MB-PLS-DA) was performed for each matrix.

PLS_Toolbox (version 9.2) software from Eigenvector Research Inc. (Manson, WA, USA) and the free and publicly online analysis software MetaboAnalyst 6.0 version (https://www.metaboanalyst.ca/ accessed on 9 July 2024), were used for the multivariate analysis. 

## 3. Results and Discussion

### 3.1. Volatile Organic Compound Identification

The chromatographic peak areas of the identified compounds in the samples were compared with those identified in the blank, and the compounds that showed a value higher than 5% in the blank were eliminated. This left a total of 204 and 99 VOCs in the hornet and in the external cover of their nests by GC-MS, respectively. The total identified compounds with their retention times (RT), mean normalised areas, match factors (MF), and reverse match factors (RMF) are listed in Table S1 of the Supplementary Material. The match factors are calculated by comparing all *m*/*z* ion fragments in the unknown spectrum with the ones of the reference spectrum from the NIST library. Whereas the reverse match factor compares the *m*/*z* ion fragments of the reference spectrum with the ones of the unknown spectrum and, thus, ensuring that the most important fragments are present in the unknown spectrum.

Of the total, 36 compounds were found in common in the hornets and in the outer cover of the nests. A bibliography search was carried out for each identified compound in common to find out if they came from insects, plants, contaminants, etc. Table 2 shows the compounds found in common between the hornets and the external cover of the nest for each of the colonies studied with their retention times, match factors, and reverse match factors. The X represents the presence of this compound in the hornets and in the external cover of the nest of each colony. In addition, those compounds that have been reported in the literature as compounds belonging to social insects are marked in bold. Compounds found in different plant species are shown in italics, and the Ref. column shows the references where the sources of the compounds were reported [16,18,20,21,22,27,28,29,30,31,32,33,34,35,36,37,38,39,40,41,42,43,44,45,46,47,48,49,50]. 

As can be seen in Table 2, most of those identified compounds in common were present in insects and plants, representing 66.7% and 41.7%, respectively. Those compounds that were reported in both constituted 16.7%. However, three of the total compounds in common were not found in the literature as insect and plant components.

Those common VOCs reported in plants are secondary metabolites that may have allelopathic functions. This biological function in living organisms, such as some plants, insects, and microorganisms, is based on released biochemical compounds that may have a negative or positive influence on other living organisms [51,52,53,54,55]. Allelochemicals are formed by aliphatic compounds, including lipids, fatty acids, alcohols, fatty acid esters, aromatic compounds, or terpenes, among others [54,56]. VOCs identified in this work and reported in the literature in plants belong to this group of compounds.

Many allelochemicals or mixtures of them have been described as insect-attracting compounds [51,57,58]. This means that the results obtained in this work could represent an advance in the knowledge about the interactions between the *V. velutina* hornets and the plants that the species selects for their nest construction. Studies of the negative allelopathic function of plants as pest control have been increasing in recent years due to environmental protection reasons [53,54,55]. However, there are few works that describe the attractiveness function of these allelochemicals for insects. This fact makes the information obtained on the VOCs identified in common in the four colonies have an important relevance, as they open the door to the research and development of new control methods based on attractant traps or baits containing possible species-specific compounds.

### 3.2. Multivariate Analysis

#### 3.2.1. Multiblock-Principal Component Analysis (MB-PCA)

A MB-PCA was applied to the VOCs identified in the hornets and the external cover of the nests in both solvents in order to examine possible clustering as well as outliers between the different *V. velutina* colonies.

**Figure 2:**
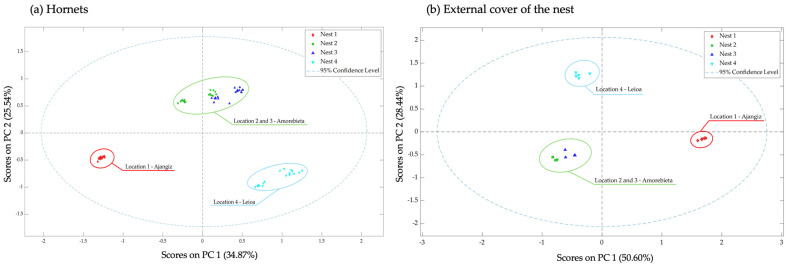
Scores plots PC1 and PC2 of the MB-PCA built with the total volatile organic compounds identified in the hornets (**a**) and the external cover of the nests (**b**) coloured by the location of the different colonies: location 1—Ajangiz (red), location 2 and 3—Amorebieta (green and dark blue), and location 4—Leioa (light blue).

Figure 2 represents the score plots of the first two components of the hornets and external cover of the nests, coloured by location of the colonies, which explain, respectively, 60.41% and 79.04% of the total variance.

The MB-PCA was able to cluster hornets and the nests according to the different colonies, as well as individuals that shared a common location. The second component (PC2) of the hornets can be considered a differentiator of the position of the nests on the map, being that Leioa and Ajangiz were the closest to the water place and Amorebieta to the inland. Whereas in the external cover of the nest analysis, there was no PC clearly differentiated.

#### 3.2.2. Multiblock-Partial Least Square Discriminatory Analysis (MB-PLS-DA)

In order to explore the variables and their correlation according to the studied colonies of *V. velutina,* a MB-PLS-DA was applied. For the model construction, the chromatographic peak areas of the identified VOCs in hornets and in the external cover of the nest were used as non-dependent variables, and the samples grouped by colonies were used as discriminant classes.

**Figure 3:**
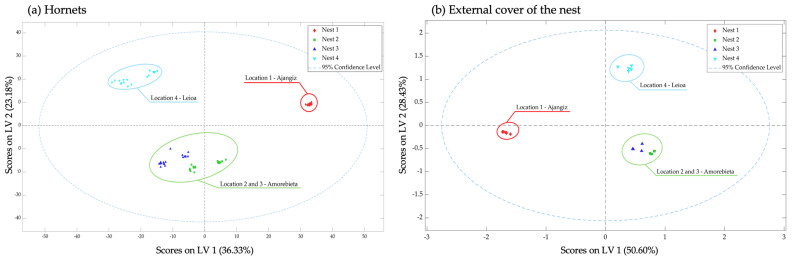
Scores plot PC1 and PC2 of the MB-PLS-DA built with the total volatile organic compounds identified in hornets of *Vespa velutina* (**a**) and the external cover of the nest (**b**) from different colonies and locations: nest 1: Ajangiz (red), nests 2 and 3: Amorebieta (green and dark blue), and nest 4: Leioa (light blue).

As can be seen in Figure 3, MB-PLS-DA was capable of slightly improving the discrimination between the colonies collected in Amorebieta, compared with the one obtained by means of MB-PCA.

With the aim of evaluating the predictive ability of the model and its statistical significance, to indicate that the fit and classification of the model were not fortuitous, it was validated by cross-validation (CV) (Venetian blind) and permutation testing (1000 iterations).

The plot of the predicted Y-values for the CV of the samples is given as Supplementary Material (Figure S1).

**Figure 4:**
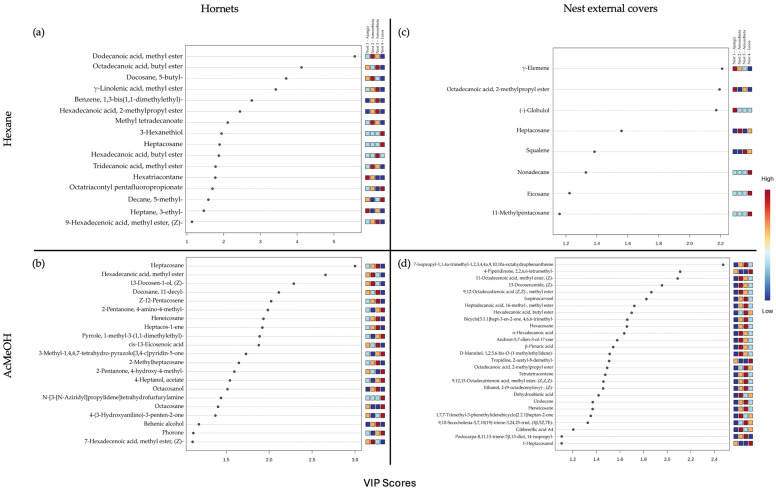
Partial least squares discriminant analysis (MB-PLS-DA) variable importance projection (VIP) scores of the VOCs identified in the analysis of hornets in hexane (**a**) and Ac:MeOH (**b**) and in the external cover of the nests in hexane (**c**) and Ac:MeOH (**d**), and the heat map of the relative concentration of each compound in the different colonies of *Vespa velutina* studied.

The next step was to explore the correlation between the VOCs identified in the hornets and in the outer cover of the nests, according to the different colonies. In this case, to obtain as much information as possible, the analyses were carried out separately for each extraction solvent. For this purpose, the variable importance projection (VIP) values were calculated for all compounds in each matrix. Those above the threshold (>1) were visualised as a heatmap (Figure 4). To keep only the compounds most closely correlated with the discrimination classes, it was decided to adjust the threshold to 1.10. For the hornet extracted in hexane and Ac:MeOH, 16 and 21 compounds exceeded the threshold, respectively. In the case of external cover of the nest, 8 and 27 compounds exceeded the threshold for the hexane and Ac:MeOH extraction, respectively.

As shown in Figure 4, the volatile organic compound profiles both in hornets and the external cover of the nest have a discriminant capacity. In these profiles, long-chain compounds are those with the higher VIP values. Among them are fatty acid esters, fatty acid alcohols, and hydrocarbons.

The compounds that exceeded the threshold VIP values were searched in the literature, and almost all were reported as compounds found in different parts of the plants and in the composition of insects [16,18,20,21,22,27,28,29,30,31,32,33,34,35,36,37,38,39,40,41,42,43,44,45,46,47,48,49,50,59,60,61,62,63,64,65,66,67,68,69,70,71]. As an exception, the compound, N-[3-[N-Aziridyl]propylidene]tetrahydrofurfurylamine, was found in the literature in *E. coli* bacteria [62].

## 4. Conclusions

VOC profiles obtained for hornets and nests using polar and non-polar solvent extraction and GC-MS have allowed identifying a total of 36 compounds in common in the hornets and in the nests of *V. velutina*. Most of these compounds have been reported in the literature as belonging to different insect and plant species. In addition, some of these compounds were found in both plants and insects. This would corroborate the transfer of chemical compounds between the nest and the hornets and vice versa.

The compounds found in common have a great relevance since they could be applied to the development of more efficient and specific control methods for this invasive species based on traps and baits containing these VOCs as attractants.

The profiles of volatile organic compounds treated by MB-PCA and MB-PLS-DA chemometric tools were able to differentiate and discriminate between the different colonies. In addition, they allowed us to observe similarities in colonies close in location.

Extraction with polar and non-polar solvents provided a great number of VOCs, expanding the knowledge about the chemical composition of hornets and nests of the species *V. velutina*. Furthermore, these compounds facilitate the discriminatory power between the different colonies, resulting in a discriminatory profile that includes not only hydrocarbons but also several other families of compounds as fatty acid esters, fatty acids, and alcohols.

Further studies on a greater number of colonies and the verification of the attractant and specific character of the VOCs found in common between the hornets and the external cover of the nests would open the possibility to develop future species-specific control methods for *V. velutina*.

## Figures and Tables

**Table 1:** GC-MS analysis conditions for the extraction of volatile organic compounds in *Vespa velutina* hornets and nest external cover.

Conditions	Parameter	Conditions
GC	Carrier gas Column Injection temperature	Helium 1 mL/min (constant flow) HP-5MS UI (30 m × 0.25 mm ID × 0.25 µm) 270 °C
	Temperature programme	Initial temp.: 50 °C for 1 min Ramp: 10 °C/min to 150 °C; 5 °C/min to 250 °C; 15 °C/min to 300 °C and hold 2 min
	Scan time	36.3 min
MS	Mode	SCAN
	*m*/*z* range	40 to 400
	Detector temperature	300 °C

**Table 2:** Volatile organic compounds identified in common in *Vespa velutina* hornets and in the external cover of the nest, their retention times, match factor, and reverse match factor. Compounds in bold are those reported in the literature belonging to social insects, and those in italics were found in plant species. Literature references in which the compounds were found are included in the Ref. column.

Compounds	RT (min)	MF (%)	RMF (%)	Nest 1	Nest 2	Nest 3	Nest 4	Ref.
**11-Methylpentacosane**	31.821	86.2	93.3		X	X		[22,27]
**11-Octadecenoic acid, methyl ester**	24.776	90.6	91.2		X	X	X	[20]
**1-Decanol, 2-hexyl-**	11.392	71.7	71.8	X	X	X	X	[33]
*1-Dodecanol, 3,7,11-trimethyl-*	8.555	79.3	81.7	X	X	X	X	[34]
*1-Heptacosanol*	33.986	84.6	86.1	X	X	X	X	[35]
**1-Nonadecene**	18.369	79.9	80.0	X	X	X	X	[16,36]
**2-Hexyl-1-octanol**	14.923	74.6	81.0	X	X	X	X	[37]
*2-Pentanone, 4-methoxy-4-methyl-*	5.201	95.0	97.0	X	X		X	[38]
*3-Penten-2-one, 4-methyl-*	3.827	92.7	92.8				X	[39]
*4-Piperidinone, 2,2,6,6-tetramethyl-*	8.144	89.3	90.0	X			X	[48]
***9,12,15-Octadecatrienoic acid, methyl ester, (Z,Z,Z)-***	25.249	90.5	91.1		X	X	X	[43,47,49]
***9,12-Octadecadienoic acid (Z,Z)-, methyl ester***	24.708	95.5	95.5	X	X	X	X	[43,47]
**9-Octadecenamide, (Z)-**	26.053	82.1	87.2	X	X	X	X	[20]
**Behenic alcohol**	31.114	79.5	83.1			X	X	[44]
*Benzene, 1,3-bis(1,1-dimethylethyl)-*	10.491	92.3	93.4		X	X		[40]
*Dodecane, 2,6,11-trimethyl-*	11.764	83.6	83.7	X	X	X	X	[41]
*Ethanol, 2-(9-octadecenyloxy)-, (Z)-*	35.229	82.2	85.4	X	X	X	X	[43]
**Heneicosane**	24.708	810	87.2	X	X	X	X	[18,21,22,29,32]
***Heptacosane***	34.197	86.1	91.5		X	X	X	[18,20,22,27,32,43,50]
**Hexacosane**	32.565	92.0	93.9	X	X	X	X	[18,21,22,29,32,50]
**Hexadecane, 2,6,11,15-tetramethyl-**	18.462	84.8	85.9	X	X	X	X	[29]
Hexadecanoic acid, 2-methylpropyl ester	25.600	85.0	86.8		X	X		n.f
**Hexadecanoic acid, butyl ester**	26.323	90.0	90.0			X		[45]
***Hexadecanoic acid, methyl ester***	21.668	95.0	95.1	X	X	X	X	[28,29,43,47]
**Hexatriacontane**	35.889	90.9	93.0	X	X			[50]
***n-Hexadecanoic acid***	22.535	94.1	95.0	X	X	X	X	[20,28,29,30,43]
**Nonadecane**	13.528	84.1	87.3				X	[16,21,29,36,39,50]
**Octacosane**	33.588	92.9	94.7	X	X	X	X	[20,22,27,29,32,50]
**Octadecane, 3-ethyl-5-(2-ethylbutyl)-**	34.155	77.9	79.4		X		X	[20]
***Octadecanoic acid***	26.074	90.6	90.9		X			[20,28,29,30,49]
Octadecanoic acid, butyl ester	29.668	86.6	86.7	X	X	X		n.f
**Pentacos-1-ene**	19.393	78.6	82.4	X	X	X	X	[18,22]
Pentacosane, 13-undecyl- *	33.787	73.8	75.3	X	X	X		[46]
**Pentatriacontene**	34.878	83.9	84.0			X		[32]
*Tetratetracontane*	35.335	84.1	84.8	X	X	X	X	[40]
***Tricosane***	35.335	84.1	84.8	X				[21,22,32,39]

n.f = not found in the literature; * Other source.

## Data Availability

The data presented in this study are available on request from the corresponding author due to privacy reasons.

## Supplementary Materials

The following supporting information can be downloaded at: https://www.mdpi.com/article/10.3390/insects15100811/s1, Table S1: Mean normalized chromatographic peak areas, retention times, match factor and reverse match factor for the volatile organic compounds identified in *Vespa velutina* hornets and in their external cover of the nest extracted in hexane and in the mixture acetone:methanol (50:50). Figure S1: Predicted Y-values for the hornet and the external cover of the nests samples with the location of the different colonies as discriminant classes for the CV: nest 1—Ajangiz (red), nests 2 and 3—Amorebieta (green and dark blue) and nest 4—Leioa (light blue). The red line is the classification threshold.

## References

[1] Zablotny J.E. (2009). Sociality. Encyclopedia of Insects.

[2] Billen J. Signal Variety and Communication in Social Insects. Proceedings of the Section Experimental and Applied Entomology-Netherlands Entomological Society.

[3] Singer T.L. (1998). Roles of Hydrocarbons in the Recognition Systems of Insects. Am. Zool..

[4] Martin S.J. (2017). The Asian Hornet: Threats, Biology & Expansion.

[5] Bunker S., Bunker S. (2019). The Asian Hornet Handbook.

[6] Chauzat M.-P., Martin S. (2009). A Foreigner in France: The Asian Hornet. Biologist.

[7] Leza M., Miranda M.Á., Colomar V. (2018). First Detection of *Vespa velutina nigrithorax* (Hymenoptera: Vespidae) in the Balearic Islands (Western Mediterranean): A Challenging Study Case. Biol. Invasions.

[8] Galartza E. (2016). Manual Para la Gestión de la Avispa Asiática (Vespa velutina).

[9] Monceau K., Bonnard O., Thiéry D. (2014). *Vespa velutina*: A New Invasive Predator of Honeybees in Europe. J. Pest Sci..

[10] Lamprecht I., Schmolz E., Schricker B. (2008). Pheromones in the Life of Insects. Eur. Biophys. J..

[11] Grüter C., Czaczkes T.J. (2019). Communication in Social Insects and How It Is Shaped by Individual Experience. Anim. Behav..

[12] Karlson P., Lüscher M. (1959). Pheromones: A New Term for a Class of Biologically Active Substances. Nature.

[13] Billen J., Morgan E.D. (1998). Pheromone Communication in Social Insects: Sources and Secretions. Pheromone Communication in Social Insects.

[14] Bruschini C., Cervo R., Stefano T. (2010). Pheromones in Social Wasps. Vitam. Horm..

[15] Pérez-De-Heredia I., Darrouzet E., Goldarazena A., Romón P., Iturrondobeitia J.C. (2017). Differentiating between Gynes and Workers in the Invasive Hornet *Vespa velutina* (Hymenoptera, Vespidae) in Europe. J. Hymenopt. Res..

[16] Cvačka J., Jiroš P., Šobotník J., Hanus R., Svatoš A. (2006). Analysis of Insect Cuticular Hydrocarbons Using Matrix-Assisted Laser Desorption/Ionization Mass Spectrometry. J. Chem. Ecol..

[17] Dani F.R. (2009). Cuticular Lipids as Semiochemicals in Paper Wasps and Other Social Insects. Ann. Zool. Fennici.

[18] Butts D.E., Camann M.A., Espelie K.E. (1995). Workers and Queens of the European Hornet *Vespa crabro* L. Have Colony-Specific Cuticular Hydrocarbon Profiles (Hymenoptera: Vespidae). Insectes Soc..

[19] Steinmetz I., Schmolz E., Ruther J. (2003). Cuticular Lipids as Trail Pheromone in a Social Wasp. Proc. Biol. Sci..

[20] Shantal Rodríguez-Flores M., Falcão S.I., Escuredo O., Carmen M., Carmen Seijo M., Vilas-Boas M. (2021). Chemical Profile from the Head of *Vespa velutina* and *V. crabro*. Apidologie.

[21] Ruther J., Sieben S., Schricker B. (2002). Nestmate Recognition in Social Wasps: Manipulation of Hydrocarbon Profiles Induces Aggression in the European Hornet. Naturwissenschaften.

[22] Tokoro M., Makino S. (2011). Colony and Caste Specific Cuticular Hydrocarbond Profiles in the Common Japanese Hornet, *Vespa analis* (Hymenoptera, Vespidae). Jpn. Agric. Res. Q..

[23] Singer T.L., Camann M.A., Espelie K.E. (1992). Discriminant Analysis of Cuticular Hydrocarbons of Social Wasp Polistes Exclamans Viereck and Surface Hydrocarbons of Its Nest Paper and Pedicel. J. Chem. Ecol..

[24] Espelie K.E., Gamboa G.J., Grudzien T.A., Bura E. (1994). Cuticular Hydrocarbons of the Paper Wasp *Polistes fuscatus*: A Search for Recognition Pheromones. J. Chem. Ecol..

[25] Lorenzi M.C., Bagnères A.G., Clément J.-L., Turillazzi S. (1997). Polistes Biglumis Bimaculatus Epicuticular Hydrocarbons and Nestmate Recognition (Hymenoptera, Vespidae). Insectes Sociaux.

[26] Quintanilla-Casas B., Bro R., Hinrich J.L., Davie-Martin C.L. (2023). Tutorial on PARADISe: PARAFAC2-Based Deconvolution and Identification System for Processing GC–MS Data (Protocol, Version 1). Protoc. Exch..

[27] Mitra A., Gadagkar R. (2014). The Dufour’s Gland and the Cuticle in the Social Wasp *Ropalidia marginata* Contain the Same Hydrocarbons in Similar Proportions. J. Insect Sci..

[28] Esteves F.G., dos Santos-Pinto J.R.A., Saidemberg D.M., Palma M.S. (2017). Using a Proteometabolomic Approach to Investigate the Role of Dufour’s Gland in Pheromone Biosynthesis in the Social Wasp *Polybia paulista*. J. Proteom..

[29] Wang Q., Zhou S.T., Wu X.M., Pang X.Q., Ni L.L., Yuan S.M., Yang Z.B., Li Y.H., Xiao H. (2022). GC-MS Analysis of *Vespa velutina auraria* Smith and Its Anti-Inflammatory and Antioxidant Activities in Vitro. Open Chem..

[30] Ishay J., Ikan A.R. (1976). Fatty Acids in the Tissues of Social Wasps (Hymenoptera Vespinae and Polistinae). Comp. Biochem. Physiol..

[31] Lecoq T., Lhomme P., Michez D., Dellicour S., Valterová I., Rasmont P. (2011). Molecular and Chemical Characters to Evaluate Species Status of Two Cuckoo Bumblebees: *Bombus barbutellus* and *Bombus maxillosus* (Hymenoptera, Apidae, Bombini). Syst. Entomol..

[32] Hu L., Vander Meer R.K., Porter S.D., Chen L. (2017). Cuticular Hydrocarbon Profiles Differentiate Tropical Fire Ant Populations (*Solenopsis geminata*, Hymenoptera: Formicidae). Chem. Biodivers..

[33] Vidhu V.V., Evans D.A. (2015). Ethnoentomological Values of *Oecophylla smaragdina* (Fabricius). Curr. Sci..

[34] Nazarudin M.F., Yasin I.S.M., Mazli N.A.I.N., Saadi A.R., Azizee M.H.S., Nooraini M.A., Saad N., Ferdous U.T., Fakhrulddin I.M. (2022). Preliminary Screening of Antioxidant and Cytotoxic Potential of Green Seaweed, *Halimeda opuntia* (Linnaeus) Lamouroux. Saudi J. Biol. Sci..

[35] Jakubska-Busse A., Czeluśniak I., Hojniak M., Myśliwy M., Najberek K. (2023). Chemical Insect Attractants Produced by Flowers of *Impatiens* spp. (Balsaminaceae) and List of Floral Visitors. Int. J. Mol. Sci..

[36] Lofqvist J., Bergstrom G. (1980). Volatile Communication Substances in Dufour’s Gland of Virgin Females and Old Queens of the Ant *Formica polyctena*. J. Chem. Ecol..

[37] Akhoundi M., Chebbah D., Elissa N., Brun S., Jan J., Lacaze I., Izri A. (2023). Volatile Organic Compounds: A Promising Tool for Bed Bug Detection. Int. J. Environ. Res. Public Health.

[38] El Bouchti M., Bourhia M., Alotaibi A., Aghmih K., Majid S., Ullah R., Salamatullah A.M., El Achaby M., Oumam M., Hannache H. (2021). *Stipa tenacissima* L.: A New Promising Source of Bioactive Compounds with Antioxidant and Anticancer Potentials. Life.

[39] Cecotti R., Carpana E., Falchero L., Paoletti R., Tava A. (2012). Determination of the Volatile Fraction of *Polygonum bistorta* L. at Different Growing Stages and Evaluation of Its Antimicrobial Activity against Two Major Honeybee (*Apis mellifera*) Pathogens. Chem. Biodivers..

[40] Yarazari S.B., Jayaraj M. (2022). GC–MS Analysis of Bioactive Compounds of Flower Extracts of *Calycopteris Floribunda* Lam.: A Multi Potent Medicinal Plant. Appl. Biochem. Biotechnol..

[41] Mustapha S., Jayanthi K.P.D., Parepely S.K., Hung Y., Vanhaelewyn L., Musa A.K. (2024). Behavioral and Electrophysiological Responses of Cabbage Aphids to Odors from Host Plants Infested by Conspecific and Heterospecific Herbivores. Arthropod Plant Interact..

[42] Rodríguez-Flores M.S., Falcão S.I., Escuredo O., Queijo L., Seijo M.C., Vilas-Boas M. (2021). Assessment of the in Vivo and in Vitro Release of Chemical Compounds from *Vespa velutina*. Molecules.

[43] Baeshen R.S., Baz M.M. (2023). Efficacy of *Acacia nilotica, Eucalyptus camaldulensis*, and *Salix safsafs* on the Mortality and Development of Two Vector-Borne Mosquito Species, *Culex pipiens* and *Aedes aegypti*, in the Laboratory and Field. Heliyon.

[44] Acosta-Estrada B.A., Reyes A., Rosell C.M., Rodrigo D., Ibarra-Herrera C.C. (2021). Benefits and Challenges in the Incorporation of Insects in Food Products. Front. Nutr..

[45] Carr A.L., Sonenshine D.E., Strider J.B., Roe R.M. (2016). Evidence of Female Sex Pheromones and Characterization of the Cuticular Lipids of Unfed, Adult Male versus Female Blacklegged Ticks, *Ixodes scapularis*. Exp. Appl. Acarol..

[46] Managamuri U., Vijayalakshmi M., Poda S., Rama Krishna Ganduri V.S., Satish Babu R. (2020). Bioactive Metabolites from *Streptomyces nanhaiensis* VSM-1: Polyphasic Taxonomy, Optimization, and Evaluation of Antimicrobial Metabolites by GC-MS Analysis. Medicinal Plants: Biodiversity, Sustainable Utilization and Conservation.

[47] Rahman M.M., Ahmad S.H., Mohamed M.T.M., Ab Rahman M.Z. (2014). Antimicrobial Compounds from Leaf Extracts of *Jatropha curcas*, *Psidium guajava*, and *Andrographis paniculata*. Sci. World J..

[48] Rlmington C., Roets G.C.S. (1937). Chemical Investigation of the Plant *Acalypha indica*. Isolation of Triacetonamine, a Cyanogenetic Glucoside and Quebrachite. Onderstepoort J. Vet. Res..

[49] Tangavelou A., Viswanathan M., Balakrishna K., Patra A. (2018). Phytochemical Analysis in the Leaves of *Chamaecrista nigricans* (Leguminosae). Pharm. Anal. Acta.

[50] Soares E.R.P., Batista N.R., Souza R.D.S., Torres V.D.O., Cardoso C.A.L., Nascimento F.S., Antonialli-Junior W.F. (2017). Variation of Cuticular Chemical Compounds in Three Species of *Mischocyttarus* (Hymenoptera: Vespidae) Eusocial Wasps. Rev. Bras. Entomol..

[51] Tóth M., Szentkirályi F., Vuts J., Letardi A., Tabilio M.R., Jaastad G., Knudsen G.K. (2009). Optimization of a Phenylacetaldehyde-Based Attractant for Common Green Lacewings (*Chrysoperla carnea* s.l.). J. Chem. Ecol..

[52] Shan Z., Zhou S., Shah A., Arafat Y., Arif Hussain Rizvi S., Shao H. (2023). Plant Allelopathy in Response to Biotic and Abiotic Factors. Agronomy.

[53] Begna T., Yali W. (2021). Review on the Role of Allelopathy in Pest Management and Crop Production. Int. J. Adv. Res. Biol. Sci..

[54] Nawaz A., Sarfraz M., Sarwar M., Farooq M. (2020). Ecological Management of Agricultural Pests through Allelopathy. Reference Series in Phytochemistry.

[55] Wato T. (2020). The Role of Allelopathy in Pest Management and Crop Production—A Review. Food Sci. Qual. Manag..

[56] Gajger I.T., Dar S.A. (2021). Plant Allelochemicals as Sources of Insecticides. Insects.

[57] Jaastad G., Larsson-Herrera S., Tasin M. (2020). Assessing Allelochemicals as Species-Specific Attractants for the Cherry Bark Tortrix, *Enarmonia formosana* (Lepidoptera: Tortricidae). Crop Prot..

[58] Bengtsson M., Jaastad G., Knudsen G., Kobro S., Bäckman A.C., Pettersson E., Witzgall P. (2006). Plant Volatiles Mediate Attraction to Host and Non-Host Plant in Apple Fruit Moth, *Argyresthia conjugella*. Entomol. Exp. Appl..

[59] Špaldoňová A., Havelcová M., Machovič V., Lapčák L. (2020). Molecular Resin Composition of Two Taxodium Taxa Growing in Different Climate Condition: Chromatographic and Spectroscopic Study. Adv. Med. Plant Res..

[60] Sosa A.A., Bagi S.H., Hameed I.H. (2016). Analysis of Bioactive Chemical Compounds of Euphorbia Lathyrus Using Gas Chromatography-Mass Spectrometry and Fourier-Transform Infrared Spectroscopy. J. Pharmacogn. Phytother..

[61] AlGabbani Q., Shater A.F., Assiri R., Assiri G.A., Assiri A.A., Makhlof R.T.M., Alsaad M.A., Alkhalil S.S., Almuhimed R.M., Almohaimeed H.M. (2023). Differential Effects of Methanolic Extracts of Clove, Ginger, Garlic and Eucalyptus Essential Oils on Anti-Parasitic Partitivities of *G. lamblia* and *E. histolytica*: An in Vitro Study. Rendiconti Lincei.

[62] Altaee N., Kadhim M.J., Hameed I.H. (2017). Characterization of Metabolites Produced by *E. coli* and Analysis of Its Chemical Compounds Using GC-MS. Int. J. Curr. Pharm. Rev. Res..

[63] Umebara I., Akutsu K., Kubo M., Iijima A., Sakurai R., Masutomi H., Ishihara K. (2024). Analysis of Fatty Acid Composition and Volatile Profile of Powder from Edible Crickets (*Acheta domesticus*) Reared on Apple By-Products. Foods.

[64] Suseem S.R., Mary Saral A. (2013). Analysis on Essential Fatty Acid Esters of Mushroom *Pleurotus eous* and Its Antibacterial Activity. Asian J. Pharm. Clin. Res..

[65] Xie Y., Isman M.B., Feng Y., Wong A. (1993). Diterpene Resin Acids: Major Active Principles in Tall Oil against Variegated Cutworm, *Peridroma saucia* (Lepidoptera: Notuidae). J. Chem. Ecol..

[66] Ángel Zavala-Sánchez M., Luis Rodríguez-Chávez J., Figueroa-Brito R., Quintana-López C.M., Moustapha Bah M., Campos-Guillén J., Amadeo Bustos-Martínez J., Zamora-Avella D., Ramos-López M.A. (2019). Bioactivity of 1-Octacosanol from *Senna crotalarioides* (Fabaceae: Caesalpinioideae) to Control *Spodoptera frugiperda* (Lepidoptera: Noctuidae). Florida Entomol..

[67] Seymen M., Kurtar E.S., Dursun A., Türkmen Ö. (2019). In Sustainable Agriculture: Assessment of Plant Growth Promoting Rhizobacteria in Cucurbitaceous Vegetable Crops. Field Crops Sustain. Manag. PGPR.

[68] Kortbeek R.W.J., van der Gragt M., Bleeker P.M. (2019). Endogenous Plant Metabolites against Insects. Eur. J. Plant Pathol..

[69] Arslan-Tontul S., Mutlu C., Candal C., Erbaş M. (2018). Microbiological and Chemical Properties of Wet Tarhana Produced by Different Dairy Products. J. Food Sci. Technol..

[70] Guillem R.M., Drijfhout F.P., Martin S.J. (2016). Species-Specific Cuticular Hydrocarbon Stability within European Myrmica Ants. J. Chem. Ecol..

[71] Zada A., Ben-Yehuda S., Dunkelblum E., Harel M., Assael F., Mendel Z. (2004). Synthesis and Biological Activity of the Four Stereoisomers of 4-Methyl-3-Heptanol: Main Component of the Aggregation Pheromone of *Scolytus amygdali*. J. Chem. Ecol..

