# Supplementary material for: Differentiation of Vespa velutina nigrithorax Colonies Using Volatile Organic Compound Profiles of Hornets and Nests
Source: Insects. 2024 Oct 16;15(10):811. doi: 10.3390/insects15100811 (PMC11508544; doi:10.3390/insects15100811)
Supplement: Supplementary file 1 [file insects-15-00811-s001.zip › insects-3188848-supplementary.pdf]

SUPPLEMENTARY MATERIAL

Table 1. Mean normalized chromatographic peak areas, retention times, match factor and reverse match factor for the volatile organic compounds identified in Vespa velutina hornets and in their external cover of the nest, extracted in hexane and in the mixture acetone:methanol (50:50).

				**Hornets**								**External cover of the nest**							
				**Hexane**				**AcMeOH**				**Hexane**				**AcMeOH**			
**Compounds**	**RT**	**MF**	**RMF**	**Nest 1**	**Nest 2**	**Nest 3**	**Nest 4**	**Nest 1**	**Nest 2**	**Nest 3**	**Nest 4**	**Nest 1**	**Nest 2**	**Nest 3**	**Nest 4**	**Nest 1**	**Nest 2**	**Nest 3**	**Nest 4**
(-)-Globulol	15.549	88.3	88.7									31095				710584			
(+-)-4-Amino-4,5-dihydro-2(3H)-furanone	4.787	70	71.5					1418802	876219	451565	1187065								
1-(3-(Cyclohexylamino)propyl)guanidine	13.062	58.8	60.1					93824	129128	15689	116657								
1,3,5-Pentanetriol, 3-methyl-	11.045	72.5	73.2																378864
1,7,7-Trimethyl-3-phenethylidenebicyclo[2.2.1]heptan-2-one	26.577	70.2	74.2													10930	196033	165413	256403
1,7-Dimethyl-4-(1-methylethyl)cyclodecane	9.468	77.4	82.3	14454	13465	12998	20117												
10-Heneicosene (c,t)	18.052	78.7	82									12001	16417	27753	27635				
10-Methylnonadecane	10.111	81.7	83.9	21223	21527	25537	31530												
11,14,17-Eicosatrienoic acid, methyl ester	35.305	78	83.8	1943232	2469365	1289596	1684425	2327317	960908	735694	899090								
11-Methylnonacosane	34.133	77.6	82.5					400733	244281	152914	285534								
11-Methylpentacosane	31.821	8.62	9.33					1358010	919739	533168	1061981				227314		1331630	2313024	
11-Octadecenoic acid, methyl ester	24.776	9.06	9.12					2171981	49859707	33857890	25505077						1106612	876499	979809
13-Docosen-1-ol, (Z)-	34.730	76.9	79.7						1007879	672178	498146								
13-Docosenamide, (Z)-	34.239	81.7	85.5														2441697	2464001	3205536
13-Heptadecyn-1-ol	23.338	78.8	79													132642	258622	385878	
13-Methylheptacosane	34.125	89.3	90.7	311900	556278	372955	586918	2955791	1429189	858912	2136606								
1-Butanamine, 2-methyl-N-(2-methylbutylidene)-	7.468	86.1	89.4					753631	453629	410315	603622								
1-Decanol, 2-hexyl-	11.392	7.17	7.18	157277	178706	194767	236413					33804	30487	37416	35408				
1-Decanol, 2-octyl-	18.944	78.9	79	501638	570876	692333	844523												
1-Docosene	14.919	81.2	82.1	381035	445193	523643	618200												
1-Dodecanol, 2-hexyl-	18.720	76.9	77.1									50872	61847	62850	56898				
1-Dodecanol, 2-methyl-, (S)-	13.582	71.3	72.6	78110	87917	102548	121180												
1-Dodecanol, 2-octyl-	31.119	84.1	85.9	353737	409583	496236	612853												

SUPPLEMENTARY MATERIAL

1-Dodecanol, 3,7,11-trimethyl-	8.555	7.93	8.17	13706	11592	11726	16745									199747	169395	232400	399434
1H-Azepine, 1-acetylhexahydro-	7.865	72.8	75.7																2358266
1-Heptacosanol	33.986	8.46	8.61	132875	158486	98331	256545									726496	1742937	1824465	1086451
1-Heptene, 2,6,6-trimethyl-	5.138	76.1	79.2	28679	19043	28685	40556												
1-Hexadecanol, 3,7,11,15-tetramethyl-	15.105	80	80.3	428893	494584	595062	694733												
1-Hexanol, 2-ethyl-	3.286	78.8	82.6									671081							
1H-Pyrazole, 3,5-bis(1,1-dimethylethyl)-	11.667	71.4	73.4					377489	399541	47989	734414								
1-Nonadecene	18.369	7.99	8	334809	386577	475272	572579					22283	26775	26559	25488				
1-Octadecene	14.631	84.1	84.8	323695	362609	430577	508668												
1-Octanol, 2,2-dimethyl-	10.504	83.9	84.2	159258	163021	140501	225064												
1-Octanol, 2-butyl-	10.449	81.7	85.6	44833	48900	47857	60913												
1-Octene, 3,7-dimethyl-	7.738	80	85.3	17785	15345	17335	25492												
1-Pentanol, 2-methyl-	7.967	75.6	76.9	49652															
1-Tetracosene	11.151	76.2	79.3	577490	631826	689464	827726												
1-Undecene, 8-methyl-	10.855	83.4	85.6	83516	96690	101004	113014												
2-(2-Methyl-propenyl)-cyclohexanone oxime	11.629	69.8	74					94918	107763	35644	247437								
2,2,4-Trimethyl-3-(3,8,12,16-tetramethyl-heptadeca-3,7,11,15-tetraenyl)-cyclohexanol	34.556	80.3	81.8													509145			
2,2,7,7-Tetramethyloctane	9.180	79.2	90.5	121109	102213	95359	150081												
2,2-Dimethyl-4-octenal	7.514	62.2	62.6					334660	245582	60464	348198								
2,2'-Trimethylenebis-1,3-dioxolane	11.371	72.3	77.5					2754128	2290144	580002	4067610								
2,3-Dimethyldecane	8.111	77.2	79.2	33991	29520	30141	44294												
2,4-Dimethyl-1-heptene	4.470	94.6	96.3	928100	743883	897241	1301480												
2,4-Dimethyldodecane	10.517	83.5	84	101405	99175	128532	141446												
2,6,10-Trimethyltridecane	10.462	87.2	88.1	206554	191557	176919	289259												
2,6-Dimethyldecane	8.191	86.9	88.1	193260	183038	176072	266962												
2-Cyclohexen-1-one, 3,6-dimethyl-6-(1-methylethyl)-	8.614	76.2	76.7					288989	279574	56118	328524								
2-Cyclopenten-1-one, 2,3-dimethyl-	8.229	82.1	85.5					578339	657870	117352	1366191								
2-Diethylamino-4-phenylthiooct-2-ene nitrile	14.259	70.8	79.7					90442	199548	4173	410843								
2-Hexyl-1-octanol	14.923	7.46	8.1	235069	276508	325035	382367					72972	60438	84458	92440				
2-Isopropyl-5-methyl-1-heptanol	18.230	78.2	79.8	120205	136526	177597	201682												

SUPPLEMENTARY MATERIAL

2-Methyl-1-undecanol	8.635	79.6	82.3	18480	16518	16430	24544												
2-Methyl-E-7-hexadecene	13.752	73.2	74.3	88087	100627	117562	137536												
2-Methylheptacosane	34.002	79.8	86.8						327660	211606	374968								
2-methyloctacosane	17.147	83	83	70121	86084	124131	113098												
2-Methyl-Z-4-tetradecene	26.848	80.5	81.7		290951	366774	449326												
2-Pentanone, 4-amino-4-methyl-	5.218	90.6	90.6					29720799	37358546		34300070								
2-Pentanone, 4-hydroxy-4-methyl-	4.926	91.6	92.7					92202734	64780711	25763391									
2-Pentanone, 4-methoxy-4-methyl-	5.201	9.5	9.7					171854	2785485		3031796					2687702	1029527	2622737	22516444
2-Pentenoic acid, 4-oxo-, methyl ester, (Z)-	6.491	67.9	87.9													242673	591540	325770	962123
2-Propen-1-amine, N,N-bis(1-methylethyl)-	8.115	78.4	78.8					559527	727111	237442	2108390								
3,3-Dimethyl-tetrahydro-pyrrolo[1,2-c]thiazol-1-one	6.368	77.9	80.6					787958	396981	81798	520728								
3,7-Diazabicyclo[3.3.1]nonane, 9,9-dimethyl-	7.895	68.8	69.9					193034	259186	48447	316680								
3-Hexanethiol	4.694	86	86.8				46766												
3-Methylheptacosane	33.990	83.8	90.9									201796	98282	412129	648987				
3-Methylhexacosane	33.152	75.3	86.9																
3-Methylpentacosane	32.138	92.4	96.6	853526	1363389	825797	1907448	193558	2098733	1213673	1913304								
3-Octanol, 2-methyl-	9.946	68.9	73.3					2197686	1780207	428401	2495869								
3-Octanol, 3-methyl-	9.189	68.7	73.4					202338	232477	74619	280110								
3-Penten-2-one, 4-methyl-	3.827	9.27	9.28					2788364	19860216	16212994	35000233								5078357
3-Trifluoroacetoxytetradecane	11.219	72.8	77.5													122782	117696	396340	362245
4(1H)-Quinolinone, octahydro-1-methyl-	9.083	68.2	72.1					192741	247635	25740	255762								
4,4-Dipropylheptane	9.574	81.6	85.1	23803	22782	22252	30695												
4H-Cyclopropa[5',6']benz[1',2':7,8]azuleno[5,6-b]oxiren-4-one, 8-(acetyloxy)-1,1a,1b,1c,2a,3,3a,6a,6b,7,8,8a-dodecahydro-3a,6b,8a-trihydroxy-2a-(hydroxymethyl)-1,1,5,7-tetramethyl-, [1ar-(1aα,1bβ,1cα,2aα,3aβ,6aα,6bα,7α,8β,8aα)]-	28.564	78.4	78.6														585526		
4-Heptanol, acetate	5.755	75.4	79.6					1397548	2967408	659226	2709957								
4-Piperidinone, 2,2,6,6-tetramethyl-	8.144	89.3	90					18355	632290	211380	480815					235350			5152816

SUPPLEMENTARY MATERIAL

5,10-Diethoxy-2,3,7,8-tetrahydro-1H,6H-dipyrrolo[1,2-a:1',2'-d]pyrazine	22.365	70.7	79.7					304142	258039	126645	194398								
5,8,11,14,17-Eicosapentaenoic acid, methyl ester, (all-Z)-	27.520	93.2	93.3	217515	514754	444427	183269	43349	761543	345958	293140								
5,8,11,14-Eicosatetraenoic acid, methyl ester, (all-Z)-	27.410	92.5	92.8					44635	222674	109051	73097								
5-Ethyl-1-nonene	7.819	77.6	81.9	332366	257338	353061	635520												
5H-Cyclopropa[3,4]benz[1,2-e]azulen-5-one, 4,9,9a-tris(acetyloxy)-3-[(acetyloxy)methyl]-1,1a,1b,4,4a,7a,7b,8,9,9a-decahydro-4a,7b-dihydroxy-1,1,6,8-tetramethyl-	33.859	76.8	77.2														462367	815183	
5-Hepten-2-one, 6-methyl-	6.918	76.2	78.3					584093	390291	159215	487837								
5-Methyl-2-propyl-1,4,4a,5,6,7,8,8a-octahydroquinoline	13.735	68.4	82.1					649654	659489	48972	1450126								
5-Undecene, (E)-	7.180	84	84.6	38038	32888	33808	49382												
7,10,13-Eicosatrienoic acid, methyl ester	34.941	75.5	79														1197295	1811843	
7,9-Di-tert-butyl-1-oxaspiro(4,5)deca-6,9-diene-2,8-dione	21.591	83.8	85.4													211762	201771	397618	375845
7-Hexadecenoic acid, methyl ester, (Z)-	21.122	91	91					39402	2226945	1853935	1077265								
7-Isopropyl-1,1,4a-trimethyl-1,2,3,4,4a,9,10,10a-octahydrophenanthrene	24.162	77.7	86														175350	184780	88112
7-Tetradecene, (E)-	10.339	78.9	79.1	103330	100538	105243	141197												
9,10-Secocholesta-5,7,10(19)-triene-3,24,25-triol, (3β,5Z,7E)-	30.738	70.5	74.8														278697	494612	453839
9,12,15-Octadecatrien-1-ol, (Z,Z,Z)-	34.954	75.9	82.6	2185273	2384637	1657703	2154515	2712321	1310656	888486	1367927								
9,12,15-Octadecatrienoic acid, 2,3-dihydroxypropyl ester, (Z,Z,Z)-	24.784	80.4	82.9													31742	241168	216093	363777
9,12,15-Octadecatrienoic acid, 2-phenyl-1,3-dioxan-5-yl ester	33.347	77.3	78.2													507549	1218456	1531774	
9,12,15-Octadecatrienoic acid, methyl ester, (Z,Z,Z)-	25.249	9.05	9.11					11812951	5985791	3061130	2469011						192645	208297	270802
9,12-Octadecadienoic acid (Z,Z)-	25.393	86.5	89.1													145019	1092431	642227	1359374
9,12-Octadecadienoic acid (Z,Z)-, methyl ester	24.708	9.55	9.55	2263303	2780529	3273502	1539970	5699987	2563882	2024662	1918607					138409	1257853	1163067	1365252
9-Hexadecenoic acid	22.822	74.1	75.1														138772	250582	
9-Hexadecenoic acid, methyl ester, (Z)-	21.080	92.9	92.9	795666	1022643	1192754	331591	15380	692728	341151	153069								
9-Octadecenamide, (Z)-	26.053	8.21	8.72	670076	1105158	1436253	1679039		501772		309785					8787706	7029597	10910971	23483469
9-Octadecenamide, N,N-dimethyl-	30.222	62.7	74.4													250804	161637	247168	279991
9-Octadecenoic acid (Z)-, methyl ester	24.763	93.5	93.6	10891208	13657712	13440994	6956717	24684382	10488492	6095273	5431988								

Acetamide, N-methyl-N-[4-[2-acetoxymethyl-1-pyrrolidyl]-2-butynyl]-	25.008	71.3	74.3														472785	85895	
Acetic acid n-octadecyl ester	26.628	89.9	93									224045			298530	868787	1104860	1631059	1808485
Acetic acid, 3,7,11,15-tetramethyl-hexadecyl ester	23.181	80	80.1	94506															
Androst-5,7-dien-3-ol-17-one	28.306	76.4	79.5														160528	103368	67906
Behenic alcohol	31.114	7.95	8.31							156889	269026					261952	206312	338776	298601
Benzenamine, N,N-dimethyl-4-[[(1-methylethyl)imino]methyl]-	10.893	74.8	83					174108	189932	20188	319220								
Benzene, 1,3-bis(1,1-dimethylethyl)-	10.491	9.23	9.34		88893	53706										26194	49517	311731	232071
Benzoic acid, 2,4-dihydroxy-3,6-dimethyl-, methyl ester	18.107	80.4	84.3													44001	2386017	142051	493724
Betulin	35.926	74.2	74.5													213314	669726	2315007	760797
Bicyclo[2.2.1]heptan-2-ol, 1,7,7-trimethyl-, (1S-endo)-	9.240	72.3	76.9														246676	113839	197785
Bicyclo[3.1.1]hept-2-ene-2-carboxaldehyde, 6,6-dimethyl-	9.671	75.6	78.8													11610	79227	207705	162109
Bicyclo[3.1.1]hept-3-en-2-one, 4,6,6-trimethyl-	9.882	76.3	81.4														129794	297564	184680
Biotin	15.130	63.9	64.4					124175	38201	93244	36735								
Butane, 2,2-dimethyl-	3.345	81.5	85.3									1644081							
Butyl tetratriacontyl ether	11.570	70.7	71	62425	59082	50742	92692												
Butylated Hydroxytoluene	14.276	76.9	79													241388	63535	203468	468905
Cholest-5-en-3-ol, 24-propylidene-, (3β)-	32.924	82.7	87.4					70055	236651	163154	97090								
cis-11-Eicosenoic acid	28.099	80.6	82.9					378391	341139	207524	248320								
cis-13-Eicosenoic acid	33.322	73.3	77.1						203443	163986	229821								
Cyclohexane, 1,2,3,4,5,6-hexaethyl-	12.267	73.8	75.7	48165	45902	40063	77108												
Cyclohexane, methyl-	3.510	90.1	91.8									479173							
Cyclohexanespiro-5'-(2',4',4'-trimethyl-2'-oxazoline)	8.258	70.2	74.3					279760	390821	99490	652334								
Cyclopropa[3,4]cyclohepta[1,2-a]naphthalene, 1,1a,1b,2,3,7b,8,9,10,10a-decahydro-5-methoxy-10-methylene-	24.031	70.7	72.9													8403	109567	93447	114961
Decane, 2,3,5,8-tetramethyl-	13.451	85.9	87.6	214607	237103	281654	318081												
Decane, 2,6,8-trimethyl-	10.250	72.9	77.3	68811	72367	74108	105079												
Decane, 2-methyl-	10.242	85.2	87.1	139327	130236	143399	196900												
Decane, 3,8-dimethyl-	9.553	81	81.8	40384	37481	36199	56461												

SUPPLEMENTARY MATERIAL

Decane, 4-methyl-	7.024	82.7	82.8	56598	48310	49030	90698												
Decane, 5-ethyl-5-methyl-	13.747	78.7	79.5	278158	317478	384510	432622												
Decane, 5-methyl-	6.986	83.1	83.1	91504		82370	121217												
Dehydroabietic acid	30.611	92.5	96.8													22587	6682450	546848	3304800
Diethyl Phthalate	16.077	96.8	96.9					3021127	2019801	1722296	6501281								
D-Mannitol, 1,2:5,6-bis-O-(1-methylethylidene)-	16.306	74.6	80.4														972846	276210	75032
Docosane	26.327	87.6	89.9																
Docosane, 11-decyl-	31.871	83.3	84.4						247884	160281	264779								
Docosane, 5-butyl-	33.918	76.5	79.1	273671	485489	264545													
Docosane, 7-hexyl-	32.040	77.4	81.6					286130	157294	108116	227900								
Docosyl octyl ether	22.044	79.7	80.7	205161	230859	302127	318522												
Dodecane	7.620	83.2	83.9	102367	87691	89139	133871												
Dodecane, 2,2,11,11-tetramethyl-	12.576	75	76.1	74388	74121	66857	114129												
Dodecane, 2,6,10-trimethyl-	10.026	85.5	86.9	275362	252343	231228	384624												
Dodecane, 2,6,11-trimethyl-	10.356	80.4	80.4	231726	209859	182924	353100					27964		57790	30854	575820	515647	1110588	1003716
Dodecane, 4,6-dimethyl-	10.149	87.2	87.5	235524	223975	250742	326744												
Dodecanoic acid	15.549	87.8	92.1					471130	108677	255157	169130								
Dodecanoic acid, methyl ester	14.458	88.8	89.5	33659	105600	84812													
Dotriacontane	34.375	79.4	82.9																
E-14-Hexadecenal	11.274	81.8	81.8	1436433	1548804	1708573	2058657												
Eicosane	22.835	84.8	84.8												177981				
Eicosane, 2-methyl-	17.511	79.7	82.3									22406	19994	46036	57402				
Eicosanoic acid, methyl ester	35.783	61.6	66.4					243733	92815	74874	47141								
Eicosen-1-ol, cis-9-	29.017	80.6	86.5					75604	666793	500151	356841								
Ethanol, 2-(9-octadecenyloxy)-, (Z)-	33.144	82.2	85.4					964282	487074	343662	538949					269169	311461	603836	225211
Gibb-3-ene-1,10-dicarboxylic acid, 2,4a,7-trihydroxy-1-methyl-8-methylene-, 1,4a-lactone, 10-methyl ester, (1α,2β,4aα,4bβ,10β)-	26.433	77	77														624935	144647	584285
Heneicosane	32.484	75.1	77	88717	108969	157476	142562		1392498	871883	1411393					369883	372771	425764	366994
Heptacos-1-ene	24.708	81	87.2						2079126	2148014	6467931								
Heptacosane	33.178	92.3	94.2				694043		1563661	1156204	1980272		1006350		730959	2195879	2001661	5240263	2764914
Heptadecane, 2,6,10,15-tetramethyl-	34.197	8.61	9.15	183291	172845	159328	272464												

SUPPLEMENTARY MATERIAL

Heptadecane, 2,6-dimethyl-	11.929	86.2	86.4	26060	23310	24453	35800												
Heptadecanoic acid, 16-methyl-, methyl ester	9.481	84.2	86.7														339508	346157	244730
Heptadecanoic acid, methyl ester	25.190	79.2	85.4	58848	88827	75380	30992	184996	60536	49601	50140								
Heptadecanoic acid, methyl ester	23.486	80.2	80.5																
Heptane, 2,2,3,3,5,6,6-heptamethyl-	10.318	77.4	79.6	92847	90979	104081	134667												
Heptane, 2,4,6-trimethyl-	6.284	83.2	90.1	35283	31744	33313	50032												
Heptane, 2,4-dimethyl-	4.254	87.6	88.2	110524	96882	108621	171915												
Heptane, 3-ethyl-	6.089	86.1	90.9	49168		45543													
Hexacosane	32.565	9.2	9.39	1286196	1485320	1557323	2173229	1298579	370382	281158	774057					3323470	2899128	4409168	2662114
Hexacosane, 9-octyl-	34.112	79.4	79.9													1221259	3218139	7350625	2633021
Hexadecanamide	26.340	86.1	86.5	843216	1487893	1747582	2073082	2092381	2118393	2275930	3194217								
Hexadecane	10.876	85.5	87.2	109161	114802	135820	156586												
Hexadecane, 2,6,10,14-tetramethyl-	17.266	84.7	84.7	113544	138588	183707	182304												
Hexadecane, 2,6,11,15-tetramethyl-	18.462	85.8	86.7	328082	388095	562808	522951					28216	15563	44355	26565				
Hexadecane, 2-methyl-	17.105	83.3	83.5	131732	146340	179607	221226												
Hexadecane, 7,9-dimethyl-	14.014	74	75.2	87644	103783	148218	134035												
Hexadecane, 7-methyl-	12.107	84.3	85.8	106279	102318	94758	159307												
Hexadecanoic acid, 10-hydroxy-, methyl ester	28.991	77.9	81.5					711103	244249	20096	110800								
Hexadecanoic acid, 14-methyl-, methyl ester	23.346	87.7	90.7					169069	190695	124260	90977								
Hexadecanoic acid, 2-methylpropyl ester	25.600	8.5	8.68		145090	275993						302288	204748	331821	327712	527391	936038	1468563	1710162
Hexadecanoic acid, butyl ester	26.323	9	9			558768						1128195	720244	1501633	2348539		3480520	7828046	6088819
Hexadecanoic acid, methyl ester	21.668	9.5	9.51	2574101	3749773	4284011	1253197		9876993	6179221	5589231					166600	916156	704895	584097
Hexatriacontane	35.889	9.09	9.3	705601	540765							373032	744570	690395	1332109				
Isopinocarveol	8.825	71.3	83.4														112540	345054	115521
Methyl 6-oxoheptanoate	11.747	71.6	72.5													189965	131299	405585	378579
Methyl dehydroabietate	28.983	82.4	90.1														853601		744182
Methyl eicosa-5,8,11,14,17-pentaenoate	28.032	93.4	93.5					1182632	449176	384106	262728								
Methyl stearate	25.220	94.3	94.6	1574971	2160644	2015908	802678	430259	6451116	4760208	2907691								
Methyl tetradecanoate	17.794	93.9	94.2	83633	162169	175123	44652	25608	1118018	785543	173926								

SUPPLEMENTARY MATERIAL

N-[3-[N-Aziridyl]propylidene]tetrahydrofurfurylamine	8.791	72.4	73.7					597263											
Naphthalene, 1,2,3,5,6,7,8,8a-octahydro-1,8a-dimethyl-7-(1-methylethenyl)-, [1R-(1α,7β,8aα)]-	13.244	86.9	90.2													286960			
n-Hexadecanoic acid	22.535	9.41	9.5	2763547	4863693	6137051	3397869									2107795	2088321	1605868	2629030
Nonadecane	13.528	8.41	8.73	110208	126059	161763	168061								290873				
Nonane, 3-methyl-5-propyl-	11.629	83.1	83.9	573119	529186	445324	853049												
Norgestrel, trimethylsilyl ether	25.338	67	70.6													153738	234368	368590	542387
n-Tridecan-1-ol	11.388	80.2	82.4	335376	303788	251181	317214												
Octacosane	33.588	9.29	9.47	6566352	8564873	7194995	12424069	444857	226671							1499091	3858543	9971084	4756552
Octacosanol	34.734	90.5	92.1							874373	2227718								
Octadecanal, 2-bromo-	32.548	68.6	69.1					165957	100672	80926	99199								
Octadecanamide	30.015	84	84.7					462353	668557	583013	1071758								
Octadecane	16.449	83.2	83.6	63055	62917	89880	82057												
Octadecane, 1-iodo-	32.320	83.8	87.5	593623	897973	611302	1294137												
Octadecane, 2-methyl-	15.435	84.4	86.3	33865	42303	51981	54054												
Octadecane, 3-ethyl-5-(2-ethylbutyl)-	34.155	7.79	7.94						314320							1410108	2057182	3320729	1514101
Octadecane, 6-methyl-	14.661	70.6	76.7													211054	171145	550288	687231
Octadecanoic acid	26.074	9.06	9.09	1950162	3156641	3769478	1929503	12652639	6852821	7841293	6796254						663582		
Octadecanoic acid, 2-methylpropyl ester	29.562	89.3	92.1									293276		1036765			2385565	4993125	4451408
Octadecanoic acid, butyl ester	29.668	8.66	8.67	268389	240974	429443						605755	491427	942100	1224976	2353748	1772618	3279468	2793244
Octane, 2,3,3-trimethyl-	11.625	77.5	78.7	82952	82553	79304	129511												
Octane, 3,3-dimethyl-	6.897	83.6	85.1	651815	617186	554123	871085												
Octane, 5-ethyl-2-methyl-	7.531	85.2	85.5	145531	127157	131971	193714												
Octanenitrile, 2-methylene-	8.381	65.3	66.5					1214490	1478709	237666	1811659								
Octanoic acid, 7-oxo-	8.347	70.6	71.3													212016			1320508
Octatriacontyl pentafluoropropionate	35.047	79.3	81.8	233004	223159		361793												
Octyl tetracosyl ether	23.131	79.8	81.2	295198	303153	403177	464457												
Oleic Acid	28.099	80.4	82.7					537754	471725	289777									
Oleic acid, eicosyl ester	35.427	71.3	72.1													467702	798390	1233556	1141886
Oxalic acid, 2-ethylhexyl pentyl ester	5.814	68.3	72.7					1337114	810727	381852	1315597								
Oxazole, 5-ethyl-2-methyl-4-benzoyl-	15.193	65.9	66					302128	313477	52495	322916								

Palmitoleic acid	22.086	92.8	93	646855	1047631	1163892	373679												
Pentacos-1-ene	19.393	7.86	8.24	387178	415497	511209	636014					11758	6797	14897	14066				
Pentacosane	31.406	91.3	92.9	1790366	2890583	1746364	3437076	4234766	3182024	1391663	3816088								
Pentacosane, 13-undecyl-	33.787	7.38	7.53					97292	394809	218614	352345					160523	327105	610002	
Pentadecane	11.561	86.2	86.9	253325	304894	409994	388727												
Pentadecane, 2,6,10-trimethyl-	12.056	83.9	84	224195	206711	181208	333190												
Pentadecane, 3-methyl-	12.238	84.5	85.9	123152	116552	105553	188434												
Pentatriacontene	34.878	8.39	8.4					1246088		569576	1308199							632289	
Phenol, 2,4-bis(1-methyl-1-phenylethyl)-	31.601	93.2	93.4	788172	843771	1543717	1304998												
Phenol, 2-methyl-5-(1,2,2-trimethylcyclopentyl)-, (S)-	19.206	81.9	90.9													128787	299762	263577	379583
Phorone	8.694	90.7	92					796644	776269	111428	946969								
Pimaric acid	28.915	76.3	83.6														983580		
Piperidine, 1-(1-methylpentyl)-	10.567	75	75.8					3059035	3567858	499498	1912415								
Piperidine, 1,4-dimethyl-	5.835	69.8	70.3					9778816	5032082	2195087	10213265								
Piperonyl butoxide	30.002	76.6	80					10649	18605	7362	92983								
Podocarpa-8,11,13-triene-7β,13-diol, 14-isopropyl-	28.649	82.4	86.1														51815	736349	
Propane, 1,2-dibromo-	4.681	84.2	90.5	296531	201181	171645	382138												
Propanoic acid, 2,2-dimethyl-, hexyl ester	4.191	70.4	75.3	54269	46158	53560	80891												
Pyridine, 2,4,6-trimethyl-	6.914	71.8	80					309603	436507	151052	810600								
Pyrrole, 1-methyl-3-(1,1-dimethylethyl)-	6.516	79.9	86.6					3152527	4207997	1211017	7602377								
Pyrrolidine, 1,1'-methylenebis-	8.423	73.1	86.3					599226	631110	144827	433169								
Squalene	34.434	78.7	89.9											170295	117960				
Sulfurous acid, 2-ethylhexyl nonyl ester	9.147	82.4	84.2	118565	98915	88555	143712												
Sulfurous acid, dodecyl 2-ethylhexyl ester	11.697	80	85.4	64671	60829	51919	96055												
Tetracosane, 3-ethyl-	29.626	92.3	93.9	165988	287147	169271	332012												
Tetradecanamide	22.661	87.9	88.7	195217	453822	551216	436023	502033	641708	623577	1149742								
Tetradecane	12.601	91.8	94.1	33083		83992	50618												
Tetradecane, 2,6,10-trimethyl-	22.213	83.5	85.4									136934	86275	75389	169119	213371	231795	451068	562699
Tetradecane, 3-methyl-	10.914	81.7	83	131695	120315	104575	192927												
Tetradecane, 4,11-dimethyl-	13.138	83.7	84.9	20896	31707	68920	33279												

SUPPLEMENTARY MATERIAL

Tetradecane, 4-methyl-	10.191	81.1	82.7	36411	34003	33658	51986												
Tetradecane, 6,9-dimethyl-	10.610	82.1	82.1	411340	382893	348687	588625												
Tetradecanoic acid	18.547	93.2	93.6					58572	727418	394478	143696								
Tetratetracontane	35.335	8.41	8.48	248875	424772	279499	458627									482555	923339	1504235	827306
trans-13-Octadecenoic acid	25.478	90.8	91.4													118800	1671618	788109	1837555
Trichloroacetic acid, hexadecyl ester	14.475	80.8	81.3	183072	212292	276558	307506												
Tricosane	35.335	8.41	8.48	645217	961695	1677109	703454					1430738							
Tridecane, 3-methyl-	11.895	82.4	83.9	191539	177798	154364	290761												
Tridecane, 6-propyl-	12.157	79.6	79.9	201175	184499	154020	304612												
Tridecane, 7-propyl-	12.990	79	79.2	46931	45054	46149	70957												
Tridecanoic acid, methyl ester	17.989	78.3	78.8	222733	426836	460913	107123												
Tropidine, 2-acetyl-8-demethyl-	8.115	66.5	68.5													154455			2448588
Undecane	8.229	88.2	90.4														465065	656960	827750
Undecane, 2-methyl-	11.066	83.4	83.9	91823	104013	133087	133058												
Undecane, 3,8-dimethyl-	17.832	74.4	76.9	91246	105495	137544	154236												
Undecane, 3-ethyl-	9.350	79.5	81	38811	33953	33762	49807												
Undecane, 3-methyl-	9.980	81	82.4	141689	129342	122181	197620												
Undecane, 4,8-dimethyl-	9.832	86.1	86.3	68315	68908	72316	96808												
Undecane, 4-methyl-	8.385	81	81	65217	59209	61285	91789												
Z-12-Pentacosene	31.021	93.1	93.6	518709	466436	802976	2304501		459201	521787	1954514								
β-D-Glucopyranose, 4-O-β-D-galactopyranosyl-	11.603	74.8	76.3													690136	484081	1088146	997244
β-Pimaric acid	27.955	72.2	73.4														431758	176272	173456
β-Sitosterol	34.709	85.2	88.5									134575	199833	233868	158444	564931	2297148	2078929	2550039
γ-Elemene	14.141	75.2	80.7																
γ-Linolenic acid, methyl ester	24.420	88.4	88.6	29097	65198	56903													

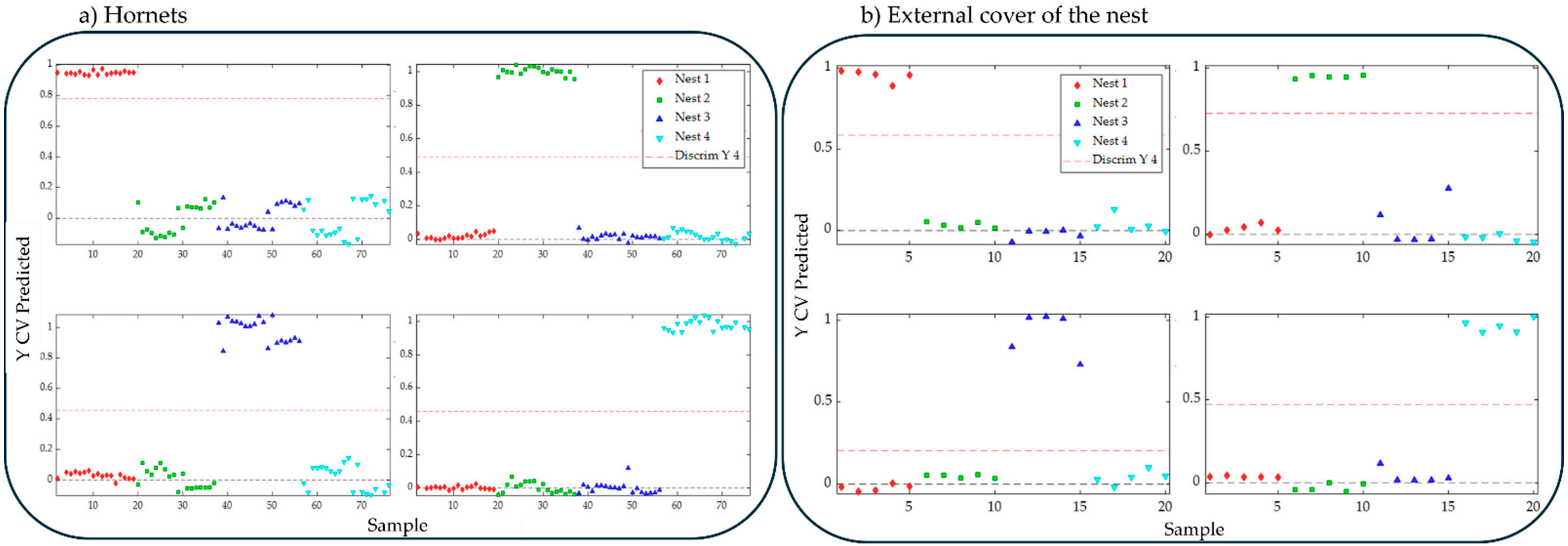

Figure 1. Predicted Y-values for the hornet and the external cover of the nests samples with the location of the different colonies as discriminant classes for the CV: nest 1 – Ajangiz (red), nests 2 and 3 – Amorebieta (green and dark blue) and nest 4 – Leioa (light blue). The red line is the classification threshold.
